# Using case study within a sequential explanatory design to evaluate the impact of specialist and advanced practice roles on clinical outcomes: the SCAPE study

**DOI:** 10.1186/1471-2288-13-55

**Published:** 2013-04-08

**Authors:** Joan G Lalor, Dympna Casey, Naomi Elliott, Imelda Coyne, Catherine Comiskey, Agnes Higgins, Kathy Murphy, Declan Devane, Cecily Begley

**Affiliations:** 1 School of Nursing and Midwifery, Trinity College Dublin, Dublin, Ireland; 2 School of Nursing & Midwifery, National University of Ireland, Galway, Ireland

**Keywords:** Case study, Specialist practice, Advanced practice, Nursing, Midwifery, Complex evaluation, Workforce

## Abstract

**Background:**

The role of the clinical nurse/midwife specialist and advanced nurse/midwife practitioner is complex not least because of the diversity in how the roles are operationalised across health settings and within multidisciplinary teams.

This aim of this paper is to use The SCAPE Study: Specialist Clinical and Advanced Practitioner Evaluation in Ireland to illustrate how case study was used to strengthen a Sequential Explanatory Design.

**Methods:**

In Phase 1, clinicians identified indicators of specialist and advanced practice which were then used to guide the instrumental case study design which formed the second phase of the larger study. Phase 2 used matched case studies to evaluate the effectiveness of specialist and advanced practitioners on clinical outcomes for service users. Data were collected through observation, documentary analysis, and interviews. Observations were made of 23 Clinical Specialists or Advanced Practitioners, and 23 matched clinicians in similar matched non-postholding sites, while they delivered care. Forty-one service users, 41 clinicians, and 23 Directors of Nursing or Midwifery were interviewed, and 279 service users completed a survey based on the components of CS and AP practice identified in Phase 1. A coding framework, and the generation of cross tabulation matrices in NVivo, was used to make explicit how the outcome measures were confirmed and validated from multiple sources. This strengthened the potential to examine single cases that seemed ‘different’, and allowed for cases to be redefined. Phase 3 involved interviews with policy-makers to set the findings in context.

**Results:**

Case study is a powerful research strategy to use within sequential explanatory mixed method designs, and adds completeness to the exploration of complex issues in clinical practice. The design is flexible, allowing the use of multiple data collection methods from both qualitative and quantitative paradigms.

**Conclusions:**

Multiple approaches to data collection are needed to evaluate the impact of complex roles and interventions in health care outcomes and service delivery. Case study design is an appropriate methodology to use when study outcomes relate to clinical practice.

## Background

Case study design has been used frequently in health and social sciences to answer complex research questions due to its flexible and pragmatic approach [1]. Case study has been described as a ‘study of the particular’ (p XI) [2] as it is allows for the study of highly context-bound phenomena with a multiplicity of variables not amenable to control. More often than not, case study is undertaken in ‘real life situations’ (p 104) [3]. It is particularly useful in organisational research [4,5] as the methods of data collection and analysis used are selected on a pragmatic basis [6] conducive to undertaking research in clinical settings. Although much has been written about case study in terms of the difference in the epistemological bases of seminal authors such as Stake and Yin, Merriam contends that “there is little consensus on what constitutes a case study or how this type of research is done”(p26) [6]. Although it is differentiated from other qualitative approaches as the focus of the research bounded by the case, this lack of certainty in definition of case study has led to some confusion, as although there is some overlap on what a case is or is not, there are significant philosophical differences between key authors. For the purpose of illustration only, let us consider three key authors in the area of case study; Merriam, Stake and Yin, in terms of where they might theoretically position case study within a qualitative-quantitative continuum. Merriam [6] contends that the single defining characteristic of case study research lies in the bounding of ‘the case’, that is giving consideration to the case as “a thing, a single entity, a unit around which there are boundaries” (p27). Case study typically utilises qualitative and quantitative data sources, selected to encapsulate the complexity of the phenomenon. Merriam [6] offers precise direction for the researcher employing a case study design in terms of using a theoretical framework to define the problem, and suggests sampling of the case (and within the case) from typical or unique examples or a range of examples to achieve maximum variation. Data are typically collected from multiple sources and can be subjected to a range of analytical strategies such as those used in, but not limited to, grounded theory, ethnography or other approaches that involve developing systems to categorise data. Although firmly grounded in a qualitative paradigm, she offered an intensely pragmatic approach to using case study in the field, and therefore (metaphorically speaking) could be located in the middle of the continuum. Stake [7] contends that the most critical role of the researcher is that of interpreter, one who constructs a view of the phenomenon through explanation and description “providing readers with good raw material for their own generalizing” (p102). Consequently, as Stake is firmly rooted in an interpretative paradigm, he could be considered to be located on the far left of the continuum. Given Stake’s position that the researcher must be “ever-reflective” (p 927) to find meanings in the data, his creative approach may have contributed to a perception that case study design is somewhat ‘elusive’ [8], as authors in nursing journals, in particular, fail to define their interpretations or offer a rationale for the approach taken [9]. Alternatively, Yin contends that a systematic approach to data collection and analysis is crucial to ensuring the methodological integrity of case study, and to demonstrate rigour, the procedural steps undertaken must be made explicit [2,8,10]. Using our continuum, Yin could be positioned on the far right, as his view also allows for the inclusion of quantitatively generated data, requires data to be collected through clearly articulated steps, integrating multiple sources of evidence to create a database of evidence in the form of narratives, documents, fieldnotes, observations etc. to increase the reliability of the case study. For this study, we consider case study as a research strategy, aligned with the position held by Yin [5,11], as given the nature of this nationwide evaluation, the large membership of the research team and multiple case sites, a structured rather than a reflective approach to data collection and analysis was required. However, the study was also influenced by the need to take a pragmatic approach [6], as data were being collected within the context of healthcare delivery.

This **aim** of this paper is to use The SCAPE Study: Specialist Clinical and Advanced Practitioner Evaluation in Ireland (present authors 2010) to illustrate how case study was used to strengthen a Sequential Explanatory Mixed Methods Design [12]. The SCAPE study evaluated the role of Clinical Nurse and Midwife Specialists (CNS/CMS) and Advanced Nurse and Midwife Practitioners (ANP/AMP), in a 3-phase study focusing on the clinical, professional and economic impact of the roles within the Irish publically funded health service. The paper will make particular reference to how case study ‘fit’ within a multiphase design, was bounded by data from phase 1 and relevant theoretical frameworks, and discuss how case study assisted in uncovering the complexity of specialist and advanced practice roles. The case study approach was deemed most appropriate for the proposed study as it examined a contemporary topic, (clinical nurse specialist and advanced nurse practitioner roles, skills, and impact), in a real life situation, (ward/unit setting), where it was difficult to extract practices from the influence of the social environment. It was important therefore, that the influence of contextual conditions on nurses’ and midwives’ practice was examined.

## Methods

### The research study: evaluation of CS and AP roles- ‘the SCAPE study’

For the past ten years, Ireland, like other countries, has seen the promulgation of specialist and advanced practice roles in nursing and midwifery. The development of this clinical career pathway has taken place against a backdrop of unparalleled health service reform. The reform measures aimed to ensure the efficient management of the health system and to consolidate health service delivery ensuring quality and value for money whilst promoting and protecting the health and welfare of the public. Changes in care delivery signalled a service requirement for clinical specialists and advanced practitioners within the multidisciplinary team, and the changing epidemiological profile of the population indicated a need for CS and AP posts to develop and proliferate into the future. Although considerable research has been undertaken both nationally and internationally evaluating the effectiveness of advanced nursing practice in many specialities, the challenges of distinguishing between tangible clinical outcomes and capturing the art of expert practice have been documented previously [13-15]. International comparisons between roles have been fraught with methodological difficulty, due to wide variations in the location of services, the organisational frameworks that dictate how practitioners work within multidisciplinary teams and the extent to which health service reform influences how the role and care pathways are operationalised. Hence, a national evaluation of these posts within the Irish health service was required if they were to continue to proliferate in line with planned reforms for service configuration and delivery [16].

### Defining case study research

Case study is a flexible research design that allows for the capture of holistic and meaningful characteristics of real life events [17-19]. Consequently it has been chosen as a favoured research approach by practitioners and policymakers [7,20]. One of the advantages of case study research is that it places emphasis on the use of multiple sources of evidence, and multiple realities, offering an opportunity to bridge paradigms [3]. Although case study can rarely be generalised, it can provide a unique understanding of the individual, organisational, social and political processes in context [11], allowing for the constraints experienced by participants in daily work to be taken into account [21]. Case study is most apt when examining complex factors that require answers [2,22,23] and when investigating nursing practice, given the multifaceted components that influence care delivery [24]. In particular, case study research is most appropriate when a: “‘how’ or ‘why’ question is being asked about a contemporary set of events over which the investigator has little or no control” (p9) [19]. Furthermore, it is an approach characterised by the use of multiple data collection methods, which provide a more “convincing and accurate” (p93) case study [17]. Although a popular methodology, case study is not without its critics, not least because of issues related to disentangling the ‘definitional morass’ (p17) [25,26]. Debates exist about the validity and generalisability of case study findings [27,28], and the fact that the two proponents of this approach, Yin and Stake, employ different terminology and epistemological positions [29]. Nevertheless, its pragmatic approach is a key strength along with its ability to facilitate ‘intensive study of one or more cases for an explicit purpose’ (p154) [28].

**Figure 1:**
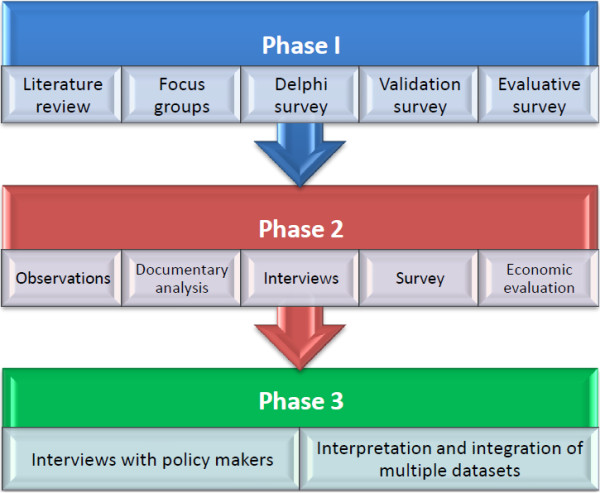
Outline of ‘The SCAPE study’: phase 2- the case study.

In this study, a sequential explanatory design (three phases) (Figure 1) was used to evaluate advanced (AP) and specialist (CS) roles across the health service seeking to determine if these roles had an impact on the clinical outcomes of patients accessing services with a post holder. Phase one involved establishing the activities and outcomes clinicians viewed to be associated with these posts through a three round Delphi method. Once established, Phase two was designed to gather data on the activities of APs and CSs and the context in which they practised. In order to undertake this evaluation, it was deemed necessary to compare the outcomes of services users receiving care from CSs and APs to those accessing a comparable service without an AP or CS working in the team. In phase two the focus was the existing state of specialist and advanced practice in Ireland; practitioners were observed and data were collected within a real-life context using multiple sources of evidence, and theoretical propositions from the literature and phase one of the study were used to guide data collection and analysis. Therefore a Yin approach to case study was deemed appropriate for several reasons:

1. The case study was ‘bounded’ by the data from phase one in relation to what clinicians deemed were the activities of postholders and clinical outcomes of these posts.

2. The sampling frame for Phase two available to the team to make comparisons was a national database of accredited post holders and the services within which they worked, held by the NCNM.

3. Cases were selected from the NCNM database of postholders to achieve maximum learning during the period of time available for the study [30]. Although we opted for a maximum variability sample in terms of location of postholder (geographical spread, urban/regional, based in acute services or community practice), case selection was ultimately limited to the availability of a matched service without a postholder for comparison. This meant that unique cases could not be sampled.

4. As it was a national study evaluating 23 (paired) services, with a large research team from two universities and four research assistants in the field, a pre-agreed theoretical/coding framework and rigorous procedures in terms of data collection and analysis (including database management) were required to ensure consistency.

5. A theoretical model of the processes and activities of advanced practice (the Logic Model) [31] was utilised as a coding framework for the analysis and integration of datasets. Practitioners’ activities were compared against a nationally agreed set of competencies for CSs and APs [32] to measure if the activities related to specialist or advanced practice.

6. Procedures to ensure internal validity included using four research assistants trained in using the tools to identify activities that represent specialist or advanced practice, regular research team meetings to ensure a shared interpretation of the data as they were being collected and analysed, analysts worked in pairs on data analysis and comparison within one of the core categories, and all findings were reviewed by one principal investigator for consistency.

7. Procedures to ensure external validity included: using independent research assistants not linked with the clinical case sites or data analysis, selection of cases to ensure generalisability of the findings within the publically funded health service in Ireland, ensuring data were categorised using a framework that would allow for international comparison (the Logic Model), extending data collection and analysis beyond the activities of the postholders to include contextual detail relevant to how the posts were operationalised.

## Results

The primary purpose of the larger research study was to understand and evaluate specialist and advanced practice in terms of the future of these posts within the health service rather than to describe the variation in how these roles were operationalised at an individual or local level. The case was deemed to be the organisation or institution where nurses or midwives worked, for example a ward or community clinic. Within this study, although 23 postholders and 23 non-postholders took part, they were at times located across a range of services provided by the same health provider. The flexibility of the case study approach ensured that the research team had access to a range of data collection strategies in order to undertake a comprehensive evaluation of what are complex, multi-faceted and intrinsically difficult to measure interventions, such as those delivered by CSs and APs [13,14,33]. The advantage to using a case study design over other triangulation methods was the continued focus on the case. As the purpose of The SCAPE Study [34] was to evaluate advanced and specialist practice roles, using case study within a sequential explanatory design allowed the research team to search for observable patterns across cases [35] at specialist and advanced practice levels, and to compare and contrast the outcomes of these roles with services without a specialist or advanced practitioner in post. This ensured that both the research assistants in the field and the research team handling the data during each phase of the study did not become distracted by the context and individuality of particular services or participants but rather allowed some degree of familiarity with the competencies demonstrated by specialist and advanced practitioners in comparison to the framework established by the NCNM for these roles.

### Case study within a sequential explanatory design

The research was based on a multiphase design (Figure 1), further details of which may be found elsewhere [36]. Ethical approval was granted by a University ethics Committee, and by all clinical sites. The case studies of postholding and matched non-postholding services comprised the second phase, and aimed to provide an in-depth exploration of the CS and AP roles with reference to the parameters identified in the literature review, focus groups with clinicians and a Delphi survey of CSs and APs undertaken in phase one (further detail is provided below).

### Case selection and context

In order to meet the objectives of the evaluation (did CS and AP roles have an impact on clinical outcomes), the cases were selected on the basis that a matched service similar to that being delivered by a CS or an AP in a non- postholding site was available for comparison. Primarily typical cases such as diabetes maternity care, anticoagulant therapy, and infectious diseases were chosen as these posts were common in the Irish health service and were also described in the literature. However, some posts were either so common as to lead to a situation whereby a service no longer existed without having a CS or AP in post, such as lactation specialists in midwifery and diabetic nurse specialist posts in general nursing. Alternatively posts were excluded as they were deemed to be so unique as to be ‘like no other’ (p 175) [37] such as fetal cardiac screening and spinal cord injury liaison. In these cases a matched non-postholding service could not be found for comparison. In keeping with an instrumental case design (the assumption being that studying CSs and APs in context is instrumental to understanding the impact of these roles on service user outcomes), the cases were purposively selected for the informational representativeness they could yield. This was determined by reviewing the national database of posts held by the National Council of Nursing and Midwifery, applying the theoretical propositions regarding specialist and advanced practice in the literature and locating a matched non-postholding service for comparison. Twenty-three postholders across nursing (general, mental health, intellectual disability, community health and children’s) and midwifery, located within acute and community settings were matched with 23 non postholding services for the case study.

### Developing a framework for case-focused analysis

In order to undertake a rigourous evaluation of specialist and advanced practice roles in Ireland, consideration was given to the need for an analytical framework to interpret and integrate the data from multiple data sources across the three phases of the study. Consideration was given to Ritchie and Spencer’s [38] framework analysis approach as it has been recognised as a valuable tool within applied policy research where, not unlike The SCAPE Study, the research question is designed to gather specific information, the sample is pre-defined (accredited practitioners registered with the NCNM), and the primary concern is to describe and interpret what is happening in context (impact of the roles). Within a framework analysis approach, a thematic framework (the second stage) is identified after the researcher has become familiarised with the transcripts/fieldnotes etc., and although themes may have been identified *a priori,* it is modified based on the themes emerging in the data. Given the nature and complexity of the SCAPE study (i.e. multiple phases, large research team, four research assistants in the field, 23 matched pairs of cases and a wide and varied range of data sources and types), a robust framework to guide data collection and analysis was essential. To achieve consistency, this framework had to be identified *a priori.* Phase one of the study aimed to identify indicators of specialist and advanced practice activities or interventions deemed to have clinical/professional impact by participants working as, or working with, CSs and APs in clinical practice. Theoretical propositions regarding the key indicators of specialist and advanced practice guided the topics for exploration in seven focus group interviews (FGIs) with five health professional groups made up of CSs, APs, Directors of Nursing/Midwifery, Medical Consultants, Staff nurses/midwives and Assistant DON/Ms, and clinical managers. Emergent data from the groups then formed the basis of an instrument that was developed and refined in a three-round Delphi study. The instrument generated in round two of the Delphi study formed the basis of the variable-orientated analysis of cases of specialist and advanced practice. Core outcomes included (but were not limited to) communication, therapeutic relationship, shared decision-making, access to care, quality of life, symptom management, use of clinical guidelines, integration of research in practice, clinical leadership, clinical and educational interventions, multidisciplinary work, continuity of care, attitudes of others to the work of the postholder and best practice in service delivery (locally (within the service), nationally (within that patient/client group in Ireland) or internationally (contribution to the field)). In addition, a theoretical framework that would allow for international comparison with the specialist and advanced practice roles as operationalised in Ireland when compared with other countries where posts have been established for longer, was required.

### Developing a framework to collect data from multiple sources in the field

It has been acknowledged in previous work on specialist, advanced practice and nurse consultant roles that these posts were developed with the intention of having a positive impact on patient/client outcomes. The underpinning assumptions to support the potential positive impact of these roles on clinical outcomes such as the clinical competencies of the individual practitioner and education of practitioners to master’s level and beyond, should be associated with the delivery of evidence based care leading to improved quality and efficiency in health care delivery. However, the actual clinical impact of these roles has been notoriously difficult to measure statistically [33]. Therefore, as an alternative to focussing on clinical outcome measures alone, Schultz et al. [39] suggested that the clinical significance of the outcome attributed to the postholder requires consideration. Guest et al. [14] contend that the impact of postholders is not limited to clinical outcomes, but rather that their activities in terms of the development of new services and the provision of clinical leadership for their colleagues may have an indirect impact on care. Consequently, if the focus were to remain on measuring clinical outcomes exclusively, the perceived professional outcomes associated with the role would fail to be captured. Gerrish et al. [13] proposed a framework of indicators of impact to take account of those with both clinical and professional significance such as symptomatology, quality of life, social significance and social validity.

Phase one of the study (literature review, focus groups and Delphi study) allowed for the identification of indicators and activities to populate these constructs and guide data collection during the observation periods within the case study. For example, core outcomes included but are not limited to: therapeutic relationship, shared decision-making, access to care, appropriateness of referral, patient/client satisfaction with information given, use of clinical guidelines by postholder, development of a new intervention, multidisciplinary teamwork, and other professionals’ knowledge level (e.g. improved understanding of clinical and social issues, patient needs/family experience). Given the size and complexity of the study, six members of the research team were involved in managing the case study, and training and supporting the four research assistants who were immersed across 23 postholding and 23 matched non postholding areas to collect data (Table 1).

**Table 1:** Case study sample

**Discipline/profession**	**Areas with postholders**	**Areas without postholders**
General nursing	9	9
Midwifery	3	3
Intellectual disability nursing	2	2
Mental health nursing	5	5
Children’s nursing	2	2
Community health	2	2
Total	23	23

Four hours of observations were conducted with each of 23 Clinical Specialists or Advanced Practitioners and 23 clinicians providing a service in similar matched non-postholding sites. Forty-one service users, 41 clinicians, and 23 Directors of Nursing or Midwifery were interviewed about their experiences of receiving care from, or working with, CS/APs or matched clinicians. Service users (n = 279) also completed a survey based on the components of CS and AP practice identified in Phase 1. Given the variation that was likely to be found in the roles, the context in which practitioners were working and the location of the service, a framework that could be applied in practice to ensure consistency in data collection and analysis was required. Bryant-Lukosius and DiCenso [40] developed ‘The PEPPA Framework’ to guide the implementation of advanced practice roles. The process involves identification of the service user group, the goal of the post, the need for a new model of care and key outcomes of the role. As the role is developed and implemented, policies and guidelines are developed and the education, support and resources that are required to support the role are determined. Each postholder in this study would have developed a job description along the lines of the PEPPA Framework in order for the post to be approved and accredited; therefore data relating to the goal of the post, expected outcomes etc. would be available to the team. Bryant-Lukosius developed the PEPPA Framework further, now known as ‘The Logic Model’ [41], in order that newly developed roles could be evaluated subsequently, taking account of both short and long-term outcomes.

Using the logic model, the postholder’s practice was thus evaluated under four main core categories: clinical practice, clinical leadership, professional leadership, and research. In this study, the indicators that represented the activities undertaken by the postholder in each of the core categories were based on the Round 2 instrument from the Delphi study. For example, in the category of clinical practice, examples of *autonomous* practice were sought and described; for research, examples of *leading* nursing and midwifery research were recorded, and so forth. The team also used the Logic Model to take account of the situation in which the postholder was working and the input (resources) required for them to carry out their role to maximum effect. An example in terms of clinical practice relating to the development of a therapeutic relationship might be that a private space/office would be required for that particular encounter, the absence of which might have a negative influence on the outcome. If the activity related to research and implementation of evidence-based practice, then access to guidelines, information databases and protected time to undertake research should form part of the structures required to support the role. Although data from Phase One generated the indicators of activities/outputs related to the role, the team took additional steps to ensure the quality of the case study within the overall sequential explanatory design. Given the volume of data, computer assisted qualitative data analysis software (CAQDAS) was required for data management and analysis. NVivo Version 8 (QSR International 2009) was selected as the package of choice, and the Logic Model [31] was used to set up the project database. Data were classified based on the following criteria: postholder/non-postholder, specialist or advanced practice, community/hospital location (each case site had a unique study identifier), discipline, service, data type (interview, observation etc.) and data source (service user, member of the multidisciplinary team etc.). Four level one codes were assigned based on the four categories from the Logic model- clinical practice, clinical leadership, professional leadership and research and audit. The use of the framework focussed data collection and analysis without limiting the richness of the data as the research assistants were asked to look for examples of specialist and advanced practice as compared with the NCNM competencies, and were not asked to look for examples of specific practices. Within each category, data were further analysed to identify evidence of activities of the postholder that demonstrated achievement (at the level of specialist or advanced practice) of the competencies defined by the NCNM. For example, within the category of research and audit, clinical specialists are required to audit their service, whereas advanced practitioners, in addition to audit, are required to lead and support research within their specialist area. From a pragmatic perspective, the case study approach also allowed for data to be gathered on facilitators and barriers to implementing the role; for example, it was noted during the periods of observation of postholders in practice that undertaking audit is more difficult for specialists (or other clinicians) working in a community as opposed to a hospital setting as data are collected manually rather than electronically.

### Steps to enhance data quality and consistency of coding decisions within the team

In order to ensure validity, robustness and comprehensiveness of the framework for data collection, data analysis and coding levels used, four external experts were requested to test it based on the initial data collected. Each expert took a sample of data and analysed it using the framework to verify if the coding matrix generated using the Logic Model [41] based on data from Phase One was appropriate, and to evaluate if the attributes in the matrix were identified in the data. This was found to be the case. In addition, the experts added to the attributes within the four first level codes, based on their sample analysis. This process of expert review, with minor additions to the activities identified in Phase One, confirmed the suitability of the framework for analysis. All narrative data (interview transcripts, field notes taken by the research assistants and synopsis of documentary evidence) from the case study were managed and analysed using the computer assisted, qualitative data analysis software NVivo Version 8 (QSR International 2009). Once all data were coded within the framework, queries were run within NVivo to contrast the findings for each of the four first level codes between postholding and non-postholding sites, in order to explore the impact of postholders on practice and service delivery. All data were analysed taking cognisance of the context in which care was delivered, and the factors that facilitate or impede practitioners in the field. The number and source of all documentary evidence collected from the sites was collated and synopsised.

### Steps to managing case study data analysis

**Figure 2:**
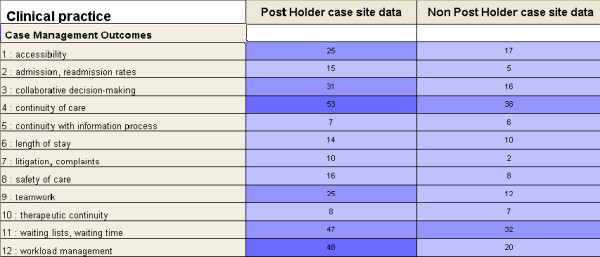
Comparison of cases in matched sites within Nvivo.

One of the key challenges in case study design is how to manage data analyses especially as the data come from multiple and diverse sources. In this case study, NVivo was used so that the case study data from the observations in clinical practice, interviews with key stakeholders such as service users, family members/carers, healthcare professionals and Directors of Nursing/ Midwifery, could be analysed and also enable researchers to compare outcomes across the case study sites with and without CSs/APs. A coding framework was constructed using the outcomes previously identified in phase 1 and within NVivo all the interview and observation data were analysed and the evidence for each outcome was extracted. In order to evaluate the outcomes of specialist and advanced practitioners, the evidence from case study sites with and without CSs/APs was cross tabulated (Figure 2). This example demonstrates how using cross tabulation matrices provided a means of managing data analysis whereby ALL the evidence relating to case management outcomes from case study data sources were presented clearly, which allowed researchers to carry out detailed comparisons for each outcome. Using a coding framework and generating cross tabulation matrices enabled the researchers to carry out a focused evaluation of the impact of the CS and AP roles on healthcare outcomes and make explicit how the outcome measures were confirmed and validated from multiple sources in clinical practice.

## Discussion

### The contribution of a case orientated approach to studying complexity within a multiphase design

As the objective of the research was to evaluate the impact of both specialist and advanced practice roles in the Irish health service, it became necessary to understand the commonalities and differences within and between the levels of specialist and advanced practice. As mentioned previously, cases were selected for their informational representativeness and comparability with matched non-postholding services, therefore a case-orientated approach to analysis was undertaken. The team compared the configuration of variables within the four primary level codes of clinical practice, clinical leadership, professional leadership and research and subunit analysis was based on whether the data were obtained from a case of a CS or an AP, or a matched service. A combination of indicators gathered from questionnaires from service users, interviews with clinicians, service users and Directors of Nursing or Midwifery, field notes recorded by the research assistants on their observations and other documentary evidence were combined within NVivo so that the strength of evidence to support a particular finding could be collated. In order to take account of the variation in context when comparing cases within, across and between CSs and APs, other variables relating to clinical significance such as educational interventions, service developments, implementation of evidence-based care and the resources available to support the role were added to the analysis. Although any one case can be considered as a unique configuration of elements, our interest lay in the comparison of outcomes between postholder and matched services. By labelling data based on whether they were generated from a postholding site or a matched non-postholding site, cross case comparisons and queries could be run within the software. With 23 postholding and 23 matched cases in the study, the large volume of data obtained inevitably led to heterogeneity [42], both in how the posts were operationalised locally, and in the resources that were available to the practitioner to fulfil the role. In addition, levels of activity that equate with advanced practice were observed amongst some clinical specialists, and consequently outcomes relating to clinical interventions were also influenced by this finding. A case orientated approach to analysis based on the presence of indicators associated with specialist and advanced practice, which were determined prior to the immersion of the research assistants in the case study sites, allowed the team to identify, for example, a case of a CS practising at an advanced level, whereas other research approaches may have analysed the configuration of variables in such a way as to consider the case as ‘deviant’, ‘unique’ or as an ‘outlier’.

Using data from multiple sources within the case study and managing the data within a computer assisted qualitative data analysis software package allowed the team to synthesise and empirically evaluate strength of evidence from multiple sources to support a particular finding. For example, the analyst could run a query to search for evidence to support a core outcome such as the effect of the CS or AP on increasing knowledge and skill of other care providers. To do this, all sources could be searched i.e. Delphi outcomes, interviews (and the number and nature of the sources), field notes of observation, documentary evidence etc. and the strength of the evidence to support the outcome measured. The impact of a CS or AP on core clinical and professional outcomes could also be analysed taking account of the support structures available for the role. For example, when postholders had protected time for audit and research activities the output was more significant. Using similar techniques, differences in care between postholding and matched services could also be evaluated.

The use of a case study approach within a multiphase study strengthened the potential to examine a single case that seemed ‘different’ to other cases, but more importantly it allowed for the case to be redefined. This was evident in the data from the clinical midwife specialist in diabetes, as her activities across each of the four categories of the Logic Model when compared to the competencies defined by the NCNM were demonstrated to be at advanced practice levels. Given that the findings of The SCAPE Study [36] might have significant policy implications for the future support and development of these roles in the Irish health service, it was critical that a methodology sufficiently flexible to take account of complexity in terms of the nature and range of data sources required to take account of multiple phenomena i.e. specialist and advanced practice, the context of service delivery (micro and macro), service user, service provider and policy maker perspectives was chosen for this evaluation study. The case study approach, through the application of a theoretical model of advanced practice, and the use of multiple data collection strategies, allowed for the rigorous examination of individual cases, the redefinition of others based on relevant causal conditions and the comparison of postholding cases with those from matched services. Even though it has been recognised that not all phenomena are amenable to measurement, some research consumers continue to remain sceptical about the veracity of the evidence produced through qualitative inquiry. In an era where the pressure of quantification continues to exist, a significant advantage to using a case study approach within a sequential explanatory design is that it allowed the research team to quantify, across the range of data sources, the level of evidence generated within the study to support particular claims regarding the effectiveness of CS and AP roles on specific clinical outcomes. For example, across the 23 postholding sites it was possible to quantify the amount of evidence that was generated from a range of data sources (service users, policy-makers, members of the multidisciplinary team, Directors of Nursing/Midwifery) and data types (interviews, surveys, observations, documentary analysis) to support the outcome of reduction in waiting times.

## Conclusions

Policy makers frequently pay particular attention to the weight of evidence given to support research claims. Consequently we would urge researchers to consider the suitability of this approach within multiphase designs to avoid losing focus on the critical question when searching for completeness in the exploration of complex phenomena, and to think about the potential to quantify the strength of evidence supporting particular outcomes especially if it is hoped the findings will influence future policy development.

### Limitations

Although this study cannot generate statistical generalisations regarding the impact of specialist and advanced practice roles worldwide, the findings can be generalised for typical posts across the Irish health service. As this study focussed on using matched comparisons to evaluate the effectiveness of the CS and AP roles, unique posts could not be evaluated. However, through the rigorous application of the Delphi method to achieve consensus on important activities and outcomes in Phase one, and the *a priori* application of the Logic Model to data collection and analysis, following an acknowledgement of the context in which the roles are operationalised, the findings from the study can be compared with similar research internationally.

## Competing interests

The authors declare that they have no competing interests.

The SCAPE study was funded by the National Council for Nursing and Midwifery (NCNM) in Ireland. The NCNM has since been dissolved and its functions have been transferred to the Nursing and Midwifery Board. Consequently, there are no financial or non-financial competing interests to declare in relation to this manuscript. The authors are funding (either personally or through their host university) the processing charge for this manuscript.

## Authors’ contributions

JL participated in the study design, data collection and analysis, project write up and drafted the manuscript. DC, NE, IC, CC, AH, and DD all participated in study design, data collection analysis and write up, CB and KM participated in study design, data collection, analysis, and were joint principal investigators with responsibility for coordinating the project and the write up. All authors read, contributed to and approved the final manuscript.

## Pre-publication history

The pre-publication history for this paper can be accessed here:

http://www.biomedcentral.com/1471-2288/13/55/prepub
